# Manipulating the position of DNA expression cassettes using location tags fused to dCas9 (Cas9-Lag) to improve metabolic pathway efficiency

**DOI:** 10.1186/s12934-020-01496-w

**Published:** 2020-12-14

**Authors:** Qianwen Xie, Siwei Li, Dongdong Zhao, Lijun Ye, Qingyan Li, Xueli Zhang, Li Zhu, Changhao Bi

**Affiliations:** 1 College of Biotechnology, Tianjin University of Science and Technology, Tianjin, 300457 P. R. China; 2 State Key Laboratory of Pathogen and Biosecurity, Beijing Institute of Biotechnology, Beijing, 100071 China; 3 Tianjin Institute of Industrial Biotechnology, Chinese Academy of Sciences, Tianjin, 300308 P. R. China; 4 Key Laboratory of Systems Microbial Biotechnology, Chinese Academy of Sciences, Tianjin, 300308 China

**Keywords:** dCas9, Complex localization, Astaxanthin, Carotenoids, *Escherichia coli*

## Abstract

**Background:**

Deactivated Cas9 (dCas9) led to significant improvement of CRISPR/Cas9-based techniques because it can be fused with a variety of functional groups to form diverse molecular devices, which can manipulate or modify target DNA cassettes. One important metabolic engineering strategy is to localize the enzymes in proximity of their substrates for improved catalytic efficiency. In this work, we developed a novel molecular device to manipulate the cellular location of specific DNA cassettes either on plasmids or on the chromosome, by fusing location tags to dCas9 (Cas9-Lag), and applied the technique for synthetic biology applications. Carotenoids like β-carotene serve as common intermediates for the synthesis of derivative compounds, which are hydrophobic and usually accumulate in the membrane compartment.

**Results:**

Carotenoids like β-carotene serve as common intermediates for the synthesis of derivative compounds, which are hydrophobic and usually accumulate in the membrane components. To improve the functional expression of membrane-bound enzymes and localize them in proximity to the substrates, Cas9-Lag was used to pull plasmids or chromosomal DNA expressing carotenoid enzymes onto the cell membrane. For this purpose, dCas9 was fused to the *E. coli* membrane docking tag GlpF, and gRNA was designed to direct this fusion protein to the DNA expression cassettes. With Cas9-Lag, the zeaxanthin and astaxanthin titer increased by 29.0% and 26.7% respectively. Due to experimental limitations, the electron microscopy images of cells expressing Cas9-Lag vaguely indicated that GlpF-Cas9 might have pulled the target DNA cassettes in close proximity to membrane. Similarly, protein mass spectrometry analysis of membrane proteins suggested an increased expression of carotenoid-converting enzymes in the membrane components.

**Conclusion:**

This work therefore provides a novel molecular device, Cas9-Lag, which was proved to increase zeaxanthin and astaxanthin production and might be used to manipulate DNA cassette location.

## Introduction

**Fig. 1:**
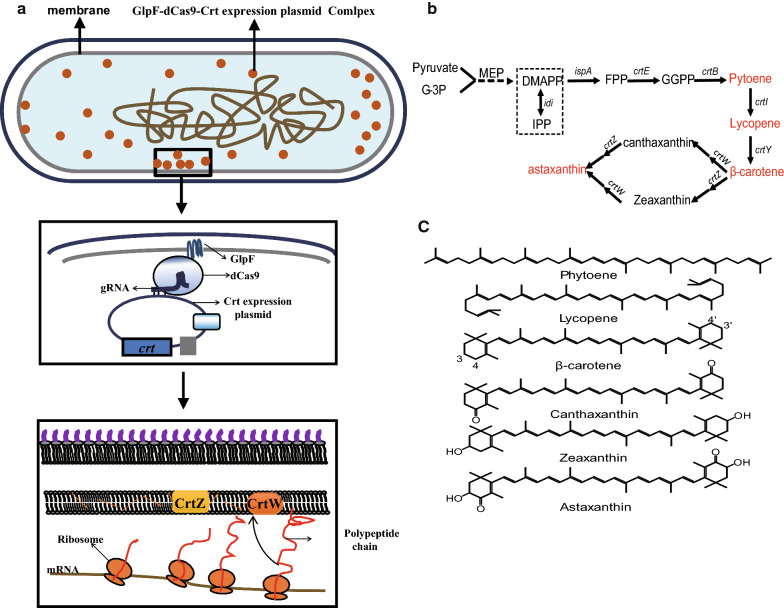
**a** Manipulate position of expression DNA cassettes strategies for improving Carotenoids production. **b** Schematic diagram of the heterologous synthesis pathway for the production of carotenoids. **c** The Molecular Formula diagram of the various carotenoids

The RNA-guided Cas9 nuclease has been developed into a powerful genome editing tool in recent years [1–3]. The Clustered Regularly Interspersed Short Palindromic Repeats CRISPR/Cas9 system is able to recognize and make double-strand breaks at target sequences based solely on a guide RNA [4, 5]. More recently, the invention of deactivated Cas9 further expanded the scope of this technique, allowing the protein to specifically bind to designed DNA sequences without inducing strand breaks. A variety of functional components were fused to dCas9 to form molecular devices, which implemented novel biological functions aimed at the target DNA, such as transcriptional blockage, epigenetic modification and gene expression regulation [4–6]. For example, the ω group of RNA polymerase was fused to dCas9 to form a complex which was guided to promoter regions of *E. coli* genes to activate transcription initiation [6, 7]. Recently, a new family of CRISPR-based genome editing methods, named base editor techniques, were developed by fusing the PmCDA1 activation-induced cytidine deaminase (AID) from sea lamprey to dCas9 to form a molecular device that can perform targeted base editing [8]. In this work, we developed a novel molecular device to manipulate the cellular position of specific DNA cassettes either on plasmids or on the chromosome, by fusing location tags to dCas9 (Cas9-Lag) and applied the technique for synthetic biology tasks (Fig. 1a).

Due to the medical and preventive value of carotenoids, metabolic engineering for the heterologous production of carotenoids has been a research hotspot for decades, and numerous novel techniques have led to advances in this field [9, 10]. In the heterologous carotenoid synthesis pathway (Fig. 1b), IPP and DMAPP are synthesized by the MVA or MEP pathway, and then sequentially converted to β-carotene by farnesyl diphosphate synthase (IspA), geranylgeranyl diphosphate synthase (CrtE), phytoene synthase (CrtB), phytoene desaturase (CrtI), and lycopeneβ-cyclase (CrtY) [11, 12]. Astaxanthin is derived from β-carotene via reactions catalyzed by the two enzymes CrtZ (3,3′-hydroxylase) and CrtW (4,4′-oxygenase) [13, 14].

Most of the intermediates upstream of the synthetic pathway are water-soluble molecules, while the downstream compounds are mostly hydrophobic (Fig. 1c) and were proved to accumulate near the membrane [15, 16]. How to efficiently organize the catalytic reactions of each step and increase the catalytic efficiency of the enzymes in the synthetic pathway is an important issue in the construction of carotenoids cell factories. Consequently, the spatial optimization of isoprenoid synthesis pathways has attracted the attention of many researchers. For instance, by targeting sesquiterpene synthases, such as valencene or amorphadiene synthase, and heterologous FDP synthase to the mitochondria, the yield of valencene and amorphadiene in *Saccharomyces cerevisiae* was increased 8- and 20-fold [17]. The entire MVA pathway was localized into the mitochondria to improve the utilization of acetyl-CoA for isoprenoid production, by dual metabolic engineering of cytoplasmic and mitochondrial, the production of isoprene in *S. cerevisiae* was respectively increased 2.1- and 1.6-fold relative to the recombinant strains with solely mitochondrial or cytoplasmic engineering [18].

In our own earlier study, we investigated the cell membrane localization of single or multiple enzymes, as well as entire metabolic pathways of carotenoid synthesis, and discussed the relationship between catalytic efficiencies and different localization strategies [19]. The focus of these strategies was to bring the enzymes to their favorable environment and in close to their substrates. However, signal peptides or membrane proteins induced localization may sometimes too complex to implement for multiple enzymes, and the formation of fusion proteins may affect the conformation and function of these proteins.

In this work, to localize plasmids or chromosomal cassettes expressing carotenoid enzymes to the cell membrane compartment, dCas9 was fused with the *E. coli* membrane docking tag GlpF [20], and gRNA was designed to direct this fusion protein to the targeted DNA cassette. By the pull of dCas9-Lag, plasmids of *crtZ* and *crtW* might be in close proximity to membrane, which bring them close to their substrates, and the functional expression of the membrane binding enzymes were increased (Fig. 1a). Finally, the impact of the localization on carotenoids production was discussed.

## Results

### The GlpF-dCas9/gRNA complex specifically binds to the target plasmid

To construct a molecular device to manipulate the cellular localization of a specific DNA cassette, GlpF was fused to the amino terminus of dCas9 with a short flexible linker (GGGS). Since dCas9 was proved to decrease the expression of genes by binding to their promoter region [21], we tested whether the GlpF-dCas9 fusion still has the specific DNA binding activity guided by gRNA, the targeting sequence of dCas9 was located upstream of the *rfp* gene.

**Fig. 2:**
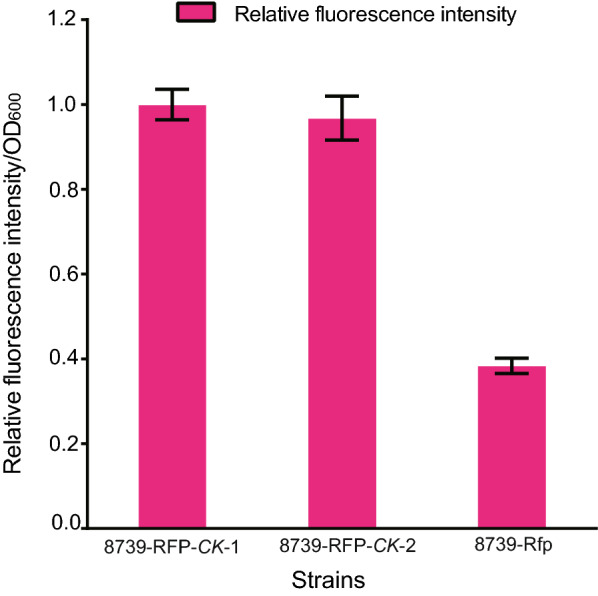
GlpF-dCas9/gRNA specifically binds to target plasmid. Expression levels of pBAD-RFP under different gene manipulation was illustrated. Compared with 8739-RFP-*CK*1 and 8739-RFP-*CK*2, the fluorescence intensity of RFP decreased significantly for the binding of GlpF-dCas9/gRNA complex to the *rfp* N terminal region. 8739-RFP(8739 co-transformed with plasmids pBAD-RFP, GlpF-dCas9 and pgRNA-RFP), 8739-RFP-*CK*-1(8739 co-transformed with plasmids pBAD-RFP, GlpF-dCas9 and pgRNA-N20), 8739-RFP-*CK*-2(8739 co-transformed with pBAD-RFP, pTrc99A-M and pgRNA-RFP)

To carry out the experiment, *E. coli* ATCC 8739 was co-transformed with the plasmids pBAD-RFP, pGlpF-dCas9 and pgRNA-RFP, which was named 8739-RFP. A pgRNA-N20 with a random non-specific 20 bp sequence was constructed and used as the control, the strain was abbreviated to 8739-RFP-*CK*1. To exclude the effect of small RNA, another control strain 8739-RFP-*CK*2 was constructed, *E. coli* ATCC 8739 was co-transformed with the plasmids pBAD-RFP, pTrc99A-M and pgRNA-RFP. The RFP fluorescence intensity of the co-transformed cells was determined after IPTG induction for 24 h. The fluorescence intensity of 8739-RFP-*CK*1 and 8739-RFP-*CK*2 were similar, while compared with 8739-RFP-*CK*1, the fluorescence intensity of 8739-RFP decreased significantly to 38.37% (Fig. 2), which indicated that the specific binding of GlpF-dCas9 to the target region guided by gRNA was functional, the binding of GlpF-dCas9 blocked the transcription of *rfp* and reduced the expression of RFP, and the small gRNA alone did not affect the expression of RFP. Thus, the fusion of GlpF to dCas9 via the GGGS linker did not affect the binding activity of dCas9, and suggested a feasible binding of GlpF-dCas9 with its target plasmid.

### Localization of the *crtW* or *crtZ* expression cassettes using dCas9-Lag for improved canthaxanthin or zeaxanthin production

The CrtW protein from *Brevundimonas sp*. SD212 has four transmembrane regions and CrtZ derived from *Pantoea agglomerans* has two transmembrane regions [22]. For the synthesis of canthaxanthin or zeaxanthin, these enzymes were expressed in *E. coli*. However, the efficiency of their targeting to the membrane compartment in heterologous hosts was unknown. We therefore attempted to use GlpF-dCas9 to localize the expression plasmid near the cell membrane and increase the functional expression of the enzymes as well as position them near their membrane-bound substrates.

The plasmids pSC101-CrtW and pSC101-CrtZ were individually introduced into the β-carotene production strain CAR025. To localize the expression cassettes of *crtW* or *crtZ* to the membrane and not affect the transcription processes of *crtZ* and *crtW* genes, the binding locus of dCas9-Lag was selected at the backbone of plasmid pSC101, Subsequently, *E. coli* CAR025 was transformed with pgRNA-pSC101bb and pGlpF-dCas9, and pgRNA-N20 with a random non-specific 20 bp sequence was introduced as a control plasmid. The resulting transformed strains were designated as CAR025-CrtW, CAR025-CrtW-*CK*, CAR025-CrtZ and CAR025-CrtZ-*CK*, respectively.

**Fig. 3:**
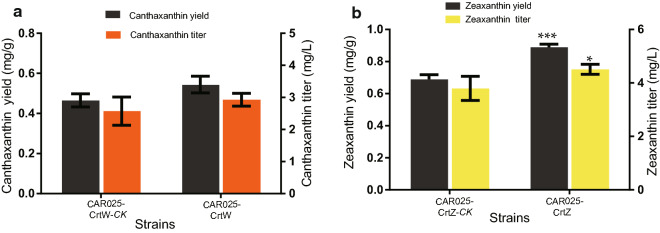
Canthaxanthin and Zeaxanthin titer and yield of strain CAR025 with different pgRNA plasmids. **a** The Canthaxanthin titer and yield of CAR025. **b** The Zeaxanthin titer and yield of strain CAR025. Three repeats were performed for each strain, and the error bars represent standard deviation. The significance of the differences was calculated by Student’s *t*-test; asterisks indicate significant differences compared with the control (*** P < 0.001; ** P < 0.01; * P < 0.05)

As shown in Fig. 3a, CAR025-CrtW produced 2.93 mg/L canthaxanthin with a specific production value of 0.54 mg/g DCW, representing an increase of 14.0 and 17.4% compared with the control strain CAR025-CrtW-*CK*, respectively (Fig. 3a). CAR025-CrtZ produced 4.51 mg/L zeaxanthin with the yield of 0.89 mg/g DCW, which was an increase of 18.8 and 29.0% compared with CAR025-CrtZ-*CK* (Fig. 3b). According to statistical analysis, the increase of zeaxanthin yield was significant (P < 0.01) and the increase of zeaxanthin titer was obvious (P < 0.05) which indicated that localizing the expression cassette of CrtZ to the membrane using dCas9-Lag improved zeaxanthin production. The increase of production was possibly caused by the increased functional expression of enzymes in the membrane and the reduced distance between enzymes and membrane-bound substrates.

Considering potential impacts on cell division and replication, cell growths and plasmid stability of CAR025-CrtZ and it’s corresponding control strain were measured. As shown in Additional file 1: Fig. S1, similar growth status was observed in both control and experiment strain, suggesting locating of the expression plasmid did not affect cell growth. Plasmids in strains CAR025-CrtZ and CAR025-CrtZ-*CK*, were gradually lost during 96 h continuously cultivation, but they were relatively stable in the first 48 h, less than 10% of plasmids were lost (Additional file 1: Fig. S2). During the culture process, with the consumption of nutrients and antibiotics, plasmids might have a tendency to be lost in strains gradually, so we collected the cells and detected the carotenoids within about 48 h. It was found both strains had similar decrease curve of their plasmid, suggesting that locating plasmids to the membrane did not affect their stability.

In order to reveal the location of the CrtW and CrtZ, RFP was linked to the C-terminal of CrtW and CrtZ. The resulting plasmids pSC101-CrtW-RFP/pSC101-CrtZ-RFP, pGlpF-dCas9 and pgRNA-pSC101bb were expressed in CAR025. Compared with Cas9-Lag inactive strains, as shown in Additional file 1: Fig. S3, CrtZ-RFP and CrtW-RFP in Cas9-Lag strain were found more towards the edges areas of the cells, but the fluorescence microscopy images could not give definite location information due to the small size of *E. coli* cells.

### Localization of the combined cassette expressing both *crtW* and *crtZ* via dCas9-Lag for improved astaxanthin production

**Fig. 4:**
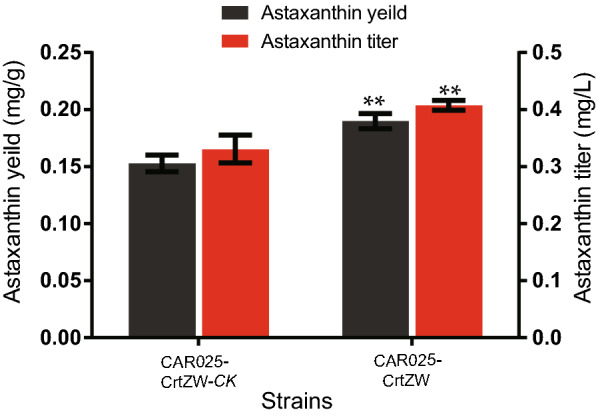
Astaxanthin titer and yield of engineered strains CAR025. Three repeats were performed for each strain, and the error bars represent standard deviation. The significance of differences was calculated by Student’s *t*-test; asterisks indicate a significant difference compared with the control (*** P < 0.001; ** P < 0.01; * P < 0.05)

The heterologous synthesis of astaxanthin from β-carotene is catalyzed by two enzymes, CrtW and CrtZ, which carry out four interchangeable reactions. The same vector pSC101 was used to express both *crtW* and *crtZ* via a constitutive promoter. To direct the plasmid carrying the synthetic pathway genes to the vicinity of the membrane compartment, pgRNA-pSC101bb and pGlpF-dcas9 were introduced into CAR025(pSC101-CrtZW) to obtain the strain CAR025-CrtZW, similar to the previous single gene experiments. CAR025-CrtZW was able to produce 0.41 mg/L astaxanthin with a specific production value of 0.19 mg/g DCW, which was 24.2 and 26.7% higher than the corresponding values of the control strain CAR025-CrtWZ-*CK* (Fig. 4). The significant astaxanthin production improvement (P < 0.01) of strain CAR025-CrtZW suggested the dCas9-Lag technique could increase astaxanthin production by localizing the combined cassette expressing the two genes *crtW* and *crtZ*.

### Protein mass spectrometry suggested increased CrtZ content in the membrane compartment

To explore the mechanism of the increase of carotenoid production in the engineered strain in which the *crt* plasmids were targeted to the membrane using GlpF-dcas9, the membrane fraction was extracted and dissolve in 10% SDS, after which the genes were amplified by PCR with corresponding primers, and the proteins were analyzed by SDS-PAGE. However, the experimental groups did not show obvious gene concentration or an increase of protein expression in the membrane.

Subsequently, protein mass spectrometry was performed on the extracted membrane protein samples to determine if the content of membrane-bound CrtW and CrtZ protein was increased by dCas9-Lag. The detailed protein mass spectrometry results of CrtW and CrtZ are marked in red in tables S2-S5. The emPAI value is used to determine the relative protein abundance, while the Sum PEP Score, Score Sequest HT and PSMs values are used to determine the protein amount indirectly. While the emPAI values of the CrtW protein in the strains CAR025-CrtW and CAR025-CrtW-*CK* were both very low, the emPAI value of the CrtZ protein in the strain CAR025-CrtZ was obviously higher than in CAR025-CrtZ-*CK*. Since mass spectral data was semi-quantitative, this result partially indicated that the expression of CrtZ protein in the membrane compartment might be improved by dCas9-Lag. And the increased amount of membrane-bound CrtZ might explain the increased zeaxanthin production by the CAR025-CrtZ strain.

According to the analysis of UniProt, CrtW from *Brevundimonas sp. SD212* has four transmembrane helixes, and CrtZ from *Pantoea agglomerans* has only two transmembrane helixes. Based on this information, maybe the membrane binding capacity of CrtW is already strong in *E. coli,* while the membrane binding capacity of CrtZ could still be improved by the Cas9-Lag technique. This result is consistent with our previous research, that the yield of zeaxanthin was significantly increased by the fusion of CrtZ with membrane tag GlpF, but the fusion of CrtW with GlpF did not significantly improve the yield of canthaxanthin [19].

### Observation of changes in the chromosome after targeting a chromosomal cassette using dCas9-Lag

Since the location of plasmids cannot be observed directly, we decided to pull the chromosomal DNA toward the cell membrane by GlpF-dCas9 for electron microscopy observation. The plasmid pgRNA-IdhAL-IdhAR was constructed with two gRNAs targeting sequences up- and down-stream of the *idh*A gene. The targeting sites were chosen at the chromosomal *idh*A sites of *E. coli* CAR005 and CAR015, which contain the crtEXYIB operon integration site. The plasmid GlpF-dCas9 was introduced into *E. coli* cells along with pgRNA-IdhAL-IdhAR.The strains with plasmid pgRNA-N20 were employed as controls.

**Fig. 5:**
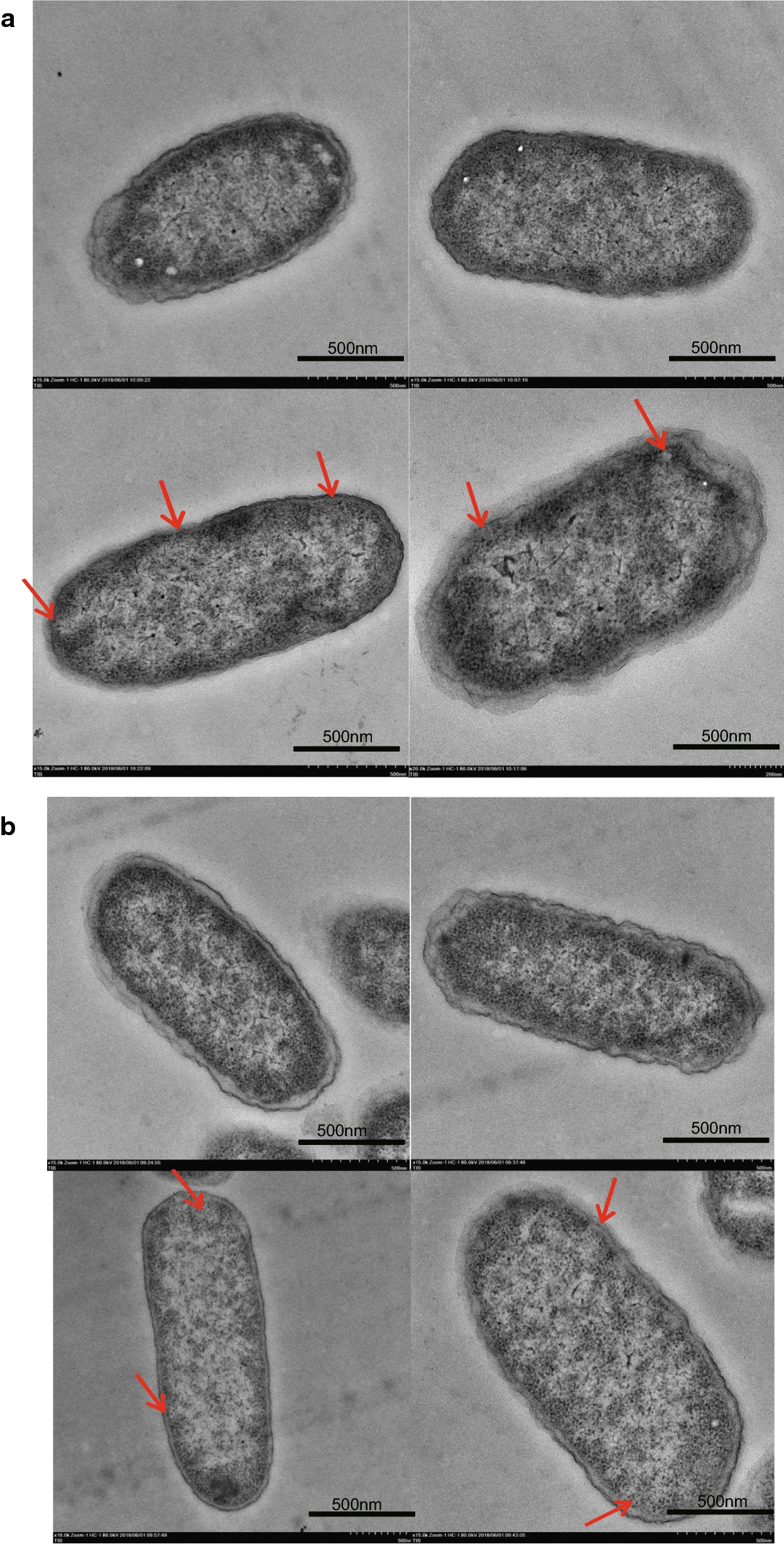
Electron microscopic pictures of the genome binding to the membrane by GlpF-dcas9 in derivatives of β-Carotene strains. **a** The change of genome distribution of CAR005. **b** The change of genome distribution of CAR015. Red arrows indicated the observed changes of membrane morphology, the distribution of the chromosome in CAR005 and CAR015 were closer to the membrane at some point as the Red arrows indicated

As illustrated in Fig. 5, cell morphology was observed in CAR005- pgRNA-IdhAL-IdhAR (Fig. 5a) and CAR015- pgRNA-IdhAL-IdhAR (Fig. 5b) compared with the control strains. Due to the interaction between GlpF-dCas9 and the target gene *idhA*, the distribution of the chromosome in the cells was closer to the membrane compartment at some points marked by red arrows. Due to experimental laminations, some of the microscopical images appeared to be vague, which somehow support the possibility that GlpF-Cas9 was functional and can mediate the localization of targeted DNA cassettes to the membrane. To determine if Cas9-Lag actually bind to IdhA gene and perturb the gene expression, we checked the transcriptional level of IdhA by RT-PCR. The relative RNA transcription levels of the IdhA fragments in CAR005 and CAR015 carrying dCas9-Lag were 14.10% and 8.58% of the parent strains, respectively, normalized by a16S internal control. This result confirmed the binding of Cas9-Lag to the IdhA gene loci.

## Discussion

Eukaryotes contain cell organelles, such as mitochondria and chloroplasts, in which a relatively independent environment is separated from other cell compartments. These organelles have their own DNA expression cassettes to produce enzymes and proteins that perform specific tasks within the compartment. Such a naturally evolved strategy to bring DNA expression cassettes into the cellular factory where its gene product is needed was proved to significantly increase the efficiency of the related cellular functions. However, due to their simple cellular organization, prokaryotes do not have the luxury of such independent organelles. In this work, we constructed a dCas9-Lag technique, the gene products of the DNA cassette were located in their favorable environment, and close to their substrates. To localize plasmids or chromosomal DNA expression cassettes to the membrane compartment, dCas9 was fused with the *E. coli* membrane docking tag GlpF [20], and a gRNA was designed to target the backbone region of the DNA cassettes. This was done to pull the DNA expression cassettes into the proximity of the cell membrane, so that the transcription and translation process could be carried out near the membrane and the produced enzymes directly localized in membrane.

As a class of long-chain hydrophobic terpenoids, carotenoids and their derivatives are insoluble in water and accumulate in membrane-containing cellular compartments. The results indicate that the functional expression of membrane proteins was enhanced in the virtual compartment produced by GlpF-dCas9 and the carotenoid gene expression cassette, while the enzymes were also brought close to their substrates in the membrane. Thus, a carotenoid producing quasi-compartment was established. In this work, we determined that pGlpF-dcas9 induced the localization of the single enzyme expression cassette pSC101-CrtZ, which significantly increased the titer of the product of zeaxanthin by 29.0%. Similarly, pGlpF-dcas9 induced the localization of the twin-enzyme expression cassette pSC101-CrtZW, which significantly increased the titer of astaxanthin by 26.7%. These results suggested that the dCas9-Lag-induced quasi-compartment was functional, creating a relatively concentrated environment for the carotenoid genes to be translated into enzymes, where the enzymes are close to their favored environment and substrates. With such a manipulation, we might have created a virtual organelle in *E. coli* for the production of carotenoids.

To confirm that dCas9-Lag induced the generation of the quasi-compartment, we tried to obtain evidence that dCas9-Lag had attached the DNA cassette onto the membrane. However, no PCR products specific for the DNA cassette were obtained using the extracted cellular membrane as template, which indicated that the DNA cassette is either not contained within the membrane compartment, or that the hydrophilic DNA was lost or shredded during the membrane extraction process. This is perhaps unsurprising, since the negative charge of the DNA constructs makes them incompatible with the hydrophobic membrane environment, and the interaction between dCas9-Lag and the plasmid might be weak, and was probably destroyed during cell disruption and ultracentrifugation. Thus, even if the DNA cassettes were pulled close to the membrane by dCas9-Lag, they could not be detected in the cellular membrane fraction following the membrane extraction process. On the other hand, to observe the function of dCas9-Lag directly, we pulled the chromosomal DNA toward the cell membrane using GlpF-dCas9, and the migration of the chromosome toward the cell membrane mediated by GlpF-dCas9 was observed using electron microscopy, although some of the images were vague due to experimental limitations. Furthermore, the protein produced by the GlpF-dCas9-bound DNA expression cassette was detected by mass spectrometry in the membrane compartment. These results all indicate that dCas9-Lag indeed successfully attached the DNA cassette to the membrane, which might have formed a cellular quasi-compartment beneficial for the function of the membrane-dependent carotenoid synthesis pathway.

Due to physical limitations and experimental limitations, the experiments performed were the best we could do to support our hypothesis that dCas9-Lag could pull DNA cassette to desired locations. And fortunately, these experiments gave us positive but not 100% absolute evidences. However, we think the strategy presented in this article was very novel and interesting. We hope fellow researchers might be inspired and do more and deeper research with ideas similar to dCas9-Lag, which might also be used to improve their microbial factories.

## Conclusion

A novel strategy was developed to fuse location tags to dCas9 (Cas9-Lag), and applied the technique for metabolic engineering. The zeaxanthin titer increased 29.0% when plasmid pSC101-CrtZ containing crtZ was localized to the membrane with Cas9-Lag, and the astaxanthin pathway product titer increased 26.7% with Cas9-Lag compared with control. This study established the novel molecular device Cas9-Lag and demonstrated that it might be able to manipulate the localization of DNA cassettes, which may have various future applications.

## Materials and methods

### Strains, media and growth conditions

The strains used in this study are listed in Table 1. All modified strains were derived from *E. coli* ATCC 8739. For seed cultures, single colonies were picked from plates and transferred into 15 mm × 100 mm tubes containing LB with chloramphenicol (30 mg/L), apramycin (50 mg/L) and ampicillin (100 mg/L), and incubated at 37 °C and 250 rpm overnight. The resulting seed cultures were subsequently used to inoculate 100 ml flasks containing 10 ml of fermentation medium with appropriate antibiotics to an initial optical density at 600 nm (OD_600_) of 0.05, and grown at 30 °C. IPTG was added to the culture after 3 h to a final concentration of 0.1 mM, and the cells were collected for measurement of products after 48 h of growth. The OD_600_ were measured constantly to determine the biomass concentration within 48 h using an SP-723 spectrophotometer (Spectrum SHANGHAI, China).

**Table 1:** Strains and plasmids used in this work

Strains and plasmids	Relative characteristics	Resources
Strains		
ATCC8739	Wild type	Lab collection
CAR005	ATCC 8739, M1-37::*dxs*, M1-46::*idi* M1-93::*Crt*, M1-46::*SucAB*, M1-46::*sdh*, M1-46::*talB*	Zhao et al. [11]
CAR015	CAR005, ispG-mRSL-4, ispH-mRSL-14	Lab collection
CAR025	CAR015, replacing the promoter of *crtEYIB* with Ptrc promoter	Lab collection
CAR025-CrtW	CAR025 ( pSC101-CrtW,pGlpF-dCas9, pgRNA-pSC101bb)	This work
CAR025-CrtW-*CK*	CAR025 (pSC101-CrtW,pGlpF-dCas9, pgRNA-N20)	This work
CAR025-CrtZ	CAR025 (pSC101-CrtZ,pGlpF-dCas9, pgRNA-pSC101bb)	This work
CAR025-CrtZ-*CK*	CAR025 (pSC101-CrtZ,pGlpF-dCas9, pgRNA-N20)	This work
CAR025-CrtZW	CAR025 (pSC101-CrtZW,pGlpF-dCas9, pgRNA-pSC101bb)	This work
CAR025-CrtZW-*CK*	CAR025 (pSC101-CrtZW,pGlpF-dCas9, pgRNA-N20)	This work
8739-RFP	ATCC 8739 (pBAD-RFP, pGlpF-dCas9, pgRNA-RFP)	This work
8739-RFP-*CK*1	ATCC 8739 (pBAD-RFP, pGlpF-dCas9, pgRNA-N20)	This work
8739-RFP-*CK*2	ATCC 8739 (pBAD-RFP, pTrc99A-M, pgRNA-N20)	This work
Plasmids		
pACYC184-M	cat; replace tet with lacI and Ptrc of pTrc99A-M	Zhao et al. [11]
pCas9	Cas9 expression plasmid	Zhao et al. (2016)
pSC101	low copy plasmid, ori and repA, M1-46 promoter, cat from pACYC184-M2-Pm46	Lab collection
pYL501	*crtW* and *crtZ* in pSC101	Lab collection
pCrtW	*crtW* in pSC101	This work
pCrtZ	*crtZ* in pSC101	This work
pCrtZW	*crtW* and *crtZ* in pSC101	This work
pGlpF-dCas9	*GlpF* fused with *dCas9* in pTrc99A, pMB1	This work
pgRNA-RFP	Apr, p15A, gRNA-rfp 20 bp guide sequence(catgcgtttcaaagttcgta)	This work
pgRNA-pSC101bb	Apr, p15A, gRNA-pYL501bb 20 bp guide sequence(catgtaacacctaatagaac)	This work
pgRNA-IdhAL-IdhAR	Apr, p15A, gRNA-IdhAL-gRNA-IdhAR 20 bp guide sequence L(cagtaataacagcgcgagaa) 20 bp guide sequence R(tgttgcgctaagcctgctga)	This work
pBAD-RFP	Kna, pBAD-*rfp*, repA101	Lab collection

### Plasmid construction, transformation and plasmid stability assay

All plasmids were assembled using the Golden Gate DNA assembly method [23] and are listed in Table 1. The primers used in this study are summarized in Additional file 1: Table S1. All the primers were designed with linkers for the type II restriction enzyme *Bsa*I for DNA assembly.

The backbone for the expression of the carotenoid synthesis pathway was amplified from the plasmid pSC101 and its derivative pYL501. Fragments containing *crtW* and *crtZ* were amplified from pYL501 [11]. The PCR products were subjected to DpnI digestion (10 U, 16 h, 37 °C) and gel purification before plasmid construction. The DNA fragment containing the dCas9 gene was obtained by mutating the Cas9 gene (Addgene reference number 42876). *GlpF* was amplified from the genome of *E. coli* 8739 using the primer pair GlpF-F/GlpF-R and the two fragments were assembled into the pTrc99A-M backbone. The linker between GlpF and dCas9 was GGGS [24]. Its nucleotide sequence GGCGGCGGCAGT, was divided into two parts and imbedded in the primers dCas9F and dCas9R separately.

For assembly of pgRNA plasmids, for example pgRNA-Crt, the N20 sequence (catgtaacacctaatagaac) was directly synthesized and embedded in the primers. The plasmid backbone contained the p15A replication origin and the *apr* gene. The plasmids pgRNA-RFP pgRNA-IdhAL, pgRNA-IdhAR and pgRNA-IdhAL-IdhAR were constructed in analogy to pgRNA-Crt.

Plasmids were transformed into *E. coil* cells via electroporation, fifty microliters of competent cells were mixed with two or three plasmids (about 25 ng each), which were electroporated at 2.5 kV, 200 Ω (Bio-Rad, Hercules, CA) and immediately incubated in 1 mL LB for 1 h at 37 °C, then plated on selective media.

For plasmid stability assay, overnight incubated fermentation medium was inoculated into 4 mL of fresh medium (LB containing 2% glycerol without any antibiotics), after12 h growth in 30 °C, the culture transferred again, the experiment was conducted for a total of 8 transfers (approximately 6 generations per transfer). Different batches of cultures were serially diluted, plated on LB media, after overnight growth, 100 colonies were picked and streaked on selective media, colonies on the plate were counted as plasmid carrying.

### Analysis of RFP fluorescence intensity using a microplate reader

The RFP-expressing colonies were picked and transferred into 3 mL of LB with 50 mg/L of kanamycin, 50 mg/L apramycin and 100 mg/L ampicillin, and grown at 37 °C and 250 rpm overnight. The cultures were then transferred into 15 mm × 100 mm tubes containing 3 mL of LB with the same antibiotics and grown at 37 °C and 250 rpm for 20 h. Subsequently, samples of each culture were diluted with LB four times and 200 µL of them transferred into individual wells of a 96-well plate. The blank control was 200 µL of pure LB. Fluorescence was measured at an excitation wavelength of 585 nm and an emission wavelength of 620 nm using an Infinite M200 Pro ELISA spectrometer (Tecan, Switzerland).

### Observation of cell morphology via transmission electron microscopy

After 48 h of cultivation, cells were collected, washed three times with phosphate buffered saline (PBS, pH 7.4), and fixed with 1% glutaraldehyde at 4 °C overnight. The fixed cells were resuspended in 1% osmic acid and incubated for 5 min at room temperature, and then resuspended again in fresh 1% osmic acid and incubated for 45 min at room temperature. Then the cells were dehydrated by immersion in graded ethanol solutions (50%, 70%, 80%, 90%, 95%, and 100%) for 15 min each. For embedding, cells were infiltrated by incubating for 2 h in 3:1, 1:1, and 1:3 embedding medium. Then, the cells were resuspended in pure embedding medium and incubated at room temperature overnight. The next day, cells were resuspended in fresh embedding medium and cured for 24 to 48 h at 80 °C, cut into 60 to 80 nm sections, stained with uranyl acetate and lead citrate, and examined under a Hitachi HT7700 electron microscope (Hitachi, Japan) operating at 180 kV [25, 26].

### Measurement of carotenoids produced by the cell factories

To quantify the production of astaxanthin, zeaxanthin and canthaxanthin, samples comprising 1 mL of culture broth were centrifuged at 16,200 × g for 3 min, and the cell pellet was washed with sterile water. Subsequently, 750 μL of extraction solution (acetonitrile/methanol/dichloromethane, 21:21:8, v/v/v) was added to the pellet and ultrasonicated in an ice bath for 30 min, after which the resulting lysate was centrifuged at 16,200 × g for 3 min. The resulting supernatant was collected and another 750μL of extraction solution was added to repeat the extraction [27]. The supernatants of both extraction steps were combined, centrifuged at 16,200 × g for 3 min, and filtered through a 0.22 μm organic nylon filter before analysis by high-performance liquid chromatography (HPLC) using an Agilent Series 1200 system with a variable wavelength detector set at 476 nm and a Symmetry C18 column (250 mm × 4.6 mm, 5 μm, Waters, Ireland). The carotenoid detection method was the same as reported before [11, 28]. The results represent the means of three independent experiments. The reference standards of the indicated compounds were purchased from Sigma (Sigma-Aldrich, USA). Dry cell weight (DCW) was calculated according to the empirical formula: 1 OD6_00_ = 0.323 g DCW/L.

### Extraction of cell membranes

Membrane extraction was performed according to a previously reported protocol [29]. Samples comprising 50 mL of fermentation broth were collected after 48 h of cultivation at 30 °C and resuspended in buffer (50 mM Tris–HCl pH 7.5, 150 mM NaCl). Cells were lysed using a French Press (JN-3000plus, China) at 1200 bar, and debris was removed by centrifugation at 10,000 g for 15 min. The supernatant was collected and centrifuged at 210,000 g for 1 h using a Beckman Optima L-100XP ultracentrifuge equipped with a Beckman-Coulter 41Ti rotor (Beckman-Coulter, Germany). The membrane fraction was collected at the bottom of the centrifuge tube as a pellet, and then dissolved using denaturation buffer (8 M urea, 1% DTT) and stored at −80 °C for mass spectrometry or further analysis.

### Statistical analysis and analytical techniques

The significance of differences between mean values of control and test samples was assessed using Student’s *t*-test in the open-source software suite “R” (http://cran.r-project.org/). Differences with *p* < 0.05 were regarded as obvious, *p* < 0.01 as significant, and *p* < 0.001 as very significant. The SDS-PAGE was run using the commercially purchased SurePage™ Gels (GenScript, Nanjing). The protein mass spectrometry was performed using an OrbiTrap Fusion LUMOS Tribrid Mass Spectrometer (LC–MS) (Thermo Fisher, USA) according to published methods [3, 30].

## Supplementary Information

**Additional file 1: Table S1. **The primers used in this research. **Table S2. **The protein mass spectrometry result of *E. coli *membrane. **Figure S1. **The growth curve of strains CAR025-CrtZ and CAR025-CrtZ-*CK *with in 48h of fermentation. CAR025-CrtZ (CAR025 co-transformed with plasmids pSC101-CrtZ, pGlpF-dCas9, pgRNA-pSC101bb),CAR025-CrtZ-*CK*(CAR025 co-transformed with plasmids pSC101-CrtZ, pGlpF-dCas9, pgRNA-N20). **Figure S2. **The plasmid stability assays of CAR025-CrtZ and CAR025-CrtZ-*CK*. The culture was transferred every 12 h and 8 transfers were conducted. CAR025-CrtZ (CAR025 co-transformed with plasmids pSC101-CrtZ, pGlpF-dCas9, pgRNA-pSC101bb), CAR025-CrtZ-*CK*( CAR025 co-transformed with plasmids pSC101-CrtZ,pGlpF-dCas9, pgRNA-N20). **Figure S3. **The fluorescence microscopy of cells with Cas9-Lag targeted plasmids expressing RFP fusion. a) The fluorescence microscopy images of CAR025-CrtZ-RFP and it’s control strain CAR025-CrtZ-RFP-*CK*; b) The fluorescence microscopy images of CAR025-CrtW-RFP and it’s contol strain CAR025-CrtW-RFP-*CK*.

## Data Availability

All data generated or analyzed during this study are included in this published article and its Additional file 1.
