# Supplementary material for: Manipulating the position of DNA expression cassettes using location tags fused to dCas9 (Cas9-Lag) to improve metabolic pathway efficiency
Source: Microb Cell Fact. 2020 Dec 14;19:229. doi: 10.1186/s12934-020-01496-w (PMC7737257; doi:10.1186/s12934-020-01496-w)
Supplement: Supplementary file 1 — Additional file 1: Table S1. The primers used in this research. Table S2. The protein mass spectrometry result of E. coli membrane. Figure S1. The growth curve of strains CAR025-CrtZ and CAR025-CrtZ-CK with in 48h of fermentation. CAR025-CrtZ (CAR025 co-transformed with plasmids pSC101-CrtZ, pGlpF-dCas9, pgRNA-pSC101bb),CAR025-CrtZ-CK(CAR025 co-transformed with plasmids pSC101-CrtZ, pGlpF-dCas9, pgRNA-N20). Figure S2. The plasmid stability assays of CAR025-CrtZ and CAR025-CrtZ-CK. The culture was transferred every 12 h and 8 transfers were conducted. CAR025-CrtZ (CAR025 co-transformed with plasmids pSC101-CrtZ, pGlpF-dCas9, pgRNA-pSC101bb), CAR025-CrtZ-CK( CAR025 co-transformed with plasmids pSC101-CrtZ,pGlpF-dCas9, pgRNA-N20). Figure S3. The fluorescence microscopy of cells with Cas9-Lag targeted plasmids expressing RFP fusion. a) The fluorescence microscopy images of CAR025-CrtZ-RFP and it’s control strain CAR025-CrtZ-RFP-CK; b) The fluorescence microscopy images of CAR025-CrtW-RFP and it’s contol strain CAR025-CrtW-RFP-CK. [file 12934_2020_1496_MOESM1_ESM.pdf]

Table S1 The primers used in this research

**Primers**	**Sequence (5′→ 3′)**	**Sources**
Ptrc 99A-bbF	CCAGGTCTCACTGACATGCAAGCTTGGCTGTTTTGG	This study
Ptrc 99A-bbR	CCAGGTCTCAGTCTGTTTCCTGTGTGAAATTGTTATC	This study
GlpF-F	CCAGGTCTCAAGACCATGAGTCAAACATCAACCTTGAAAGG	This study
GlpF-R	CCAGGTCTCACGCCGCCTTACAGCGAAGCTTTTTGTTCTGAAGG	This study
dcas9F	CCAGGTCTCAGGCGGCAGTATGGATAAGAAATACTCAATAGGCTTAGATATC	This study
dcas9F	CCAGGTCTCATCAGTCACCTCCTAGCTGACTCAAATC	This study
pgRNA- pSC101bbF	CCAGGTCTCAACCTAATAGAACGTTTTAGAGCTAGAAATAGCAAGTTAAAAT AAGGC	This study
pgRNA- pSC101bbF	CCAGGTCTCAAGGTGTTACATGGCTAAGATCTGACTCCATAACAGAGTACTC GC	This study
pgRNA-rfp-F	CCAGGTCTCAGTGCCATGCGTTTCAAAGTTCGTAGTTTTAGAGCTAGAAATAG C	This study
pgRNA-IdhAL-F	CCAGGTCTCAGTGCCAGTAATAACAGCGCGAGAAGTTTTAGAGCTAGAAATA GC	This study
pgRNA-IdhAR-F-	CCAGGTCTCACGGTTGTTGCGCTAAGCCTGCTGAGTTTTAGAGCTAGAAATA GC	This study
pgRNA-R	CCAGGTCTCAGCACCTGGCTAAATACGGAAGGatct	This study

Table S2 The protein mass spectrometry result of *E. coli* membrane

1. CAR025-CrtZ-*CK* membrane

1	Checked	Protein FD	Master	Accession	Descriptio	Exp. q-valu	Sum PEP S	Coverage	# Peptides	# PSMs	# Unique P	# Protein (	# AAs	MW [kDa]	calc. pI	Found in S	Modificati	emPAI	Score Sequ	# Peptides
2	FALSE	High	Master Prc	P62399	50S riboso	0	235.4364	88.82682	24	74	24	1	179	20.289	9.48	High		3454.107	263.9701	24
3	FALSE	High	Master Prc	P0A910	Outer men	0	224.5429	67.63006	28	76	28	1	346	37.178	6.42	High		222.872	264.3587	28
4	FALSE	High	Master Prc	P0ABA0	ATP syntha	0	192.5481	81.41026	25	129	25	1	156	17.253	6.24	High		2309.13	397.8808	25
5	FALSE	High	Master Prc	P0A7W1	30S riboso	0	185.7844	81.43713	15	45	15	1	167	17.592	10.11	High		344.511	169.071	15
63	FALSE	High	Master Prc	P0AFC3	NADH-quir	0	46.87002	27.21088	5	10	5	1	147	16.447	9.83	High		30.623	31.48294	5
64	FALSE	High	Master Prc	P0AFY8	Negative r	0	44.28404	46.96133	7	10	7	1	181	20.303	8.7	High		9	31.43284	7
65	FALSE	High	Master Prc	sp	008 CrtZ	0	44.00844	30.85714	9	15	9	1	175	20.166	9.99	High		55.234	41.1936	9
66	FALSE	High	Master Prc	P02413	50S riboso	0	43.44336	58.33333	11	19	11	1	144	14.971	11.18	High		14.199	59.97911	11

2.CAR025-CrtZ-*CK* membrane

	A	B	C	D	E	F	G	H	I	J	K	L	M	N	O	P	Q	R	S	T
1	Checked	Protein FD	Master	Accession	Descriptio	Exp. q-valu	Sum PEP S	Coverage	# Peptides	# PSMs	# Unique Peptid	# Protein (	# AAs	MW [kDa]	calc. pI	Found in S	Modificati	emPAI	Score Sequ	# Peptides
2	FALSE	High	Master Protein	P62399	50S riboso	0	401.396	90.50279	26	107	26	1	179	20.289	9.48	High		119376.7	377.2871	26
3	FALSE	High	Master Protein	P0A910	Outer men	0	379.7908	67.05202	31	98	31	1	346	37.178	6.42	High		1121.018	359.6518	31
4	FALSE	High	Master Protein	P0A7W1	30S riboso	0	377.5348	91.61677	18	86	18	1	167	17.592	10.11	High		9999	305.0239	18
121	FALSE	High	Master Protein	P0AEK2	3-oxoacyl-	0	38.65316	32.37705	5	9	5	1	244	25.544	7.42	High		3.394	24.15413	5
122	FALSE	High	Master Protein	P0A7U3	30S riboso	0	38.28124	58.69565	6	9	6	1	92	10.424	10.52	High		30.623	22.41668	6
123	FALSE	High	Master Protein	sp	008 CrtZ	0	38.09804	29.14286	8	14	8	1	175	20.166	9.99	High		22.714	37.4905	8
124	FALSE	High	Master Protein	P31069	Voltage-ga	0	37.99095	17.506	6	7	6	1	417	46.033	6.6	High		1.336	23.46003	6

3. CAR025-CrtW membrane

1	Checked	Protein FD	Master	Accession	Descriptio	Exp. q-valu	Sum PEP S	Coverage	# Peptides	# PSMs	# Unique P	# Protein (	# AAs	MW [kDa]	calc. pI	Found in S	Modificati	emPAI	Score Sequ	# Peptides
2	FALSE	High	Master Prc	P0A910	Outer men	0	717.5444	78.03468	37	201	37	1	346	37.178	6.42	High		39809.72	808.1846	37
3	FALSE	High	Master Prc	P0AFM6	Phage sho	0	558.1212	81.08108	34	146	34	1	222	25.477	5.49	High		23356.22	566.6931	34
4	FALSE	High	Master Prc	sp	006 CrtI	0	442.4479	58.53659	32	73	32	1	492	54.769	6.77	High		160.026	269.9953	32
5	FALSE	High	Master Prc	P0ABA6	ATP syntha	0	412.8369	73.51916	33	82	33	1	287	31.557	8.76	High		5454.595	289.8909	33
795	FALSE	High	Master Prc	P0A6F1	Carbamoy	0	3.529884	2.617801	1	1	1	1	382	41.405	6.38	High		0.145	2.594432	1
796	FALSE	High	Master Prc	P29012	Alanine ra	0	3.520137	2.52809	1	1	1	1	356	38.82	7.05	High		0.105	2.916156	1
797	FALSE	High	Master Prc	sp	009 CrtW	0	3.512862	5.327869	1	1	1	1	244	27.246	10.54	High		0.233	3.828159	1
798	FALSE	High	Master Prc	P37645	Uncharact	0	3.496482	1.457726	1	1	1	1	686	74.411	8.6	High		0.056	3.055126	1

4. CAR025-CrtW-*CK* membrane

1	Checked	Protein FD	Master	Accession	Descriptio	Exp. q-valu	Sum PEP S	Coverage	# Peptides	# PSMs	# Unique P	# Protein (	# AAs	MW [kDa]	calc. pI	Found in S	Modificati	emPAI	Score Sequ	# Peptides
2	FALSE	High	Master Prc	P0A910	Outer men	0	611.8575	79.19075	37	203	37	1	346	37.178	6.42	High		35480.34	744.9283	37
3	FALSE	High	Master Prc	P0AFM6	Phage sho	0	450.4389	88.28829	38	133	38	1	222	25.477	5.49	High		18328.81	495.1945	38
4	FALSE	High	Master Prc	sp	006 CrtI	0	376.4073	57.72358	31	70	31	1	492	54.769	6.77	High		107.264	274.2868	31
5	FALSE	High	Master Prc	P0AFG6	Dihydrolip	0	363.7696	53.58025	30	73	30	1	405	43.984	5.81	High		999	259.734	30
854	FALSE	Medium	Master Prc	P39343	HTH-type t	0.04822	0.737786	2.108434	1	1	1	1	332	37.543	6.7	High		0.179	0	1
855	FALSE	Low	Master Prc	sp	009 CrtW	0.051606	0.722849	5.327869	1	1	1	1	244	27.246	10.54	High		0.233	0	1
856	FALSE	Low	Master Prc	P27248	Aminomet	0.052692	0.721704	2.197802	1	1	1	1	364	40.121	5.57	High		0.122	1.703018	1
857	FALSE	Low	Master Prc	P52043	Propionyl-	0.058286	0.683401	1.219512	1	1	1	1	492	53.79	6.52	High		0.086	0	1

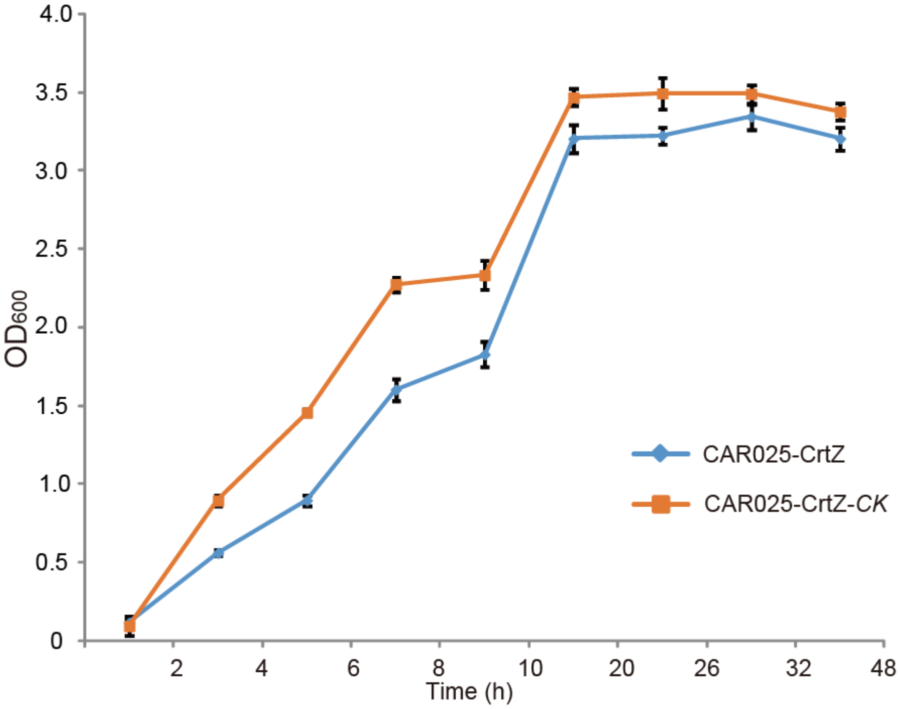

Figure S1 The growth curve of strains CAR025-CrtZ and CAR025-CrtZ-*CK* with in 48h of fermentation. CAR025-CrtZ (CAR025 co-transformed with plasmids pSC101-CrtZ, pGlpF-dCas9, pgRNA-pSC101bb),CAR025-CrtZ-*CK*(CAR025 co-transformed with plasmids pSC101-CrtZ, pGlpF-dCas9, pgRNA-N20).

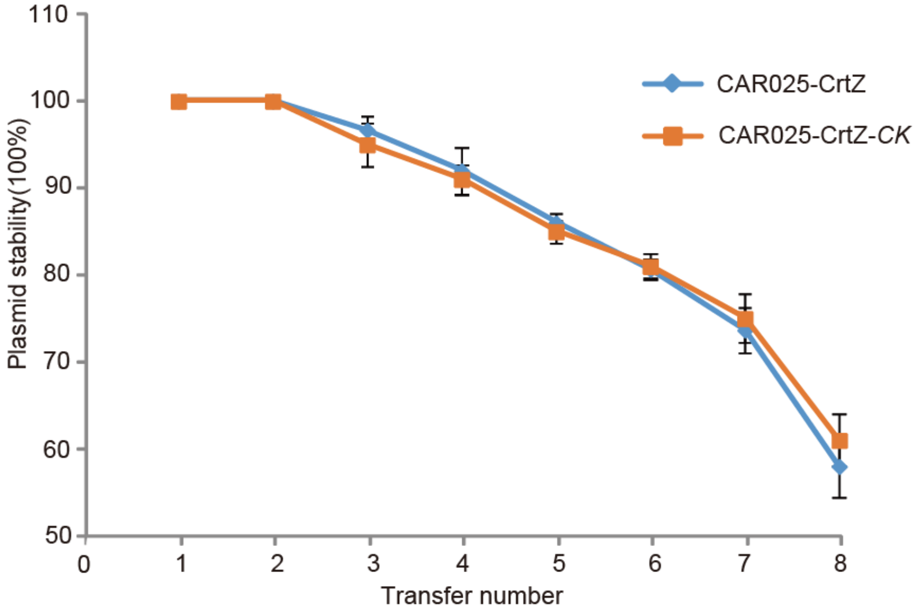

Figure S2 The plasmid stability assays of CAR025-CrtZ and CAR025-CrtZ-*CK*. The culture was transferred every 12 h and 8 transfers were conducted. CAR025-CrtZ (CAR025 co-transformed with plasmids pSC101-CrtZ, pGlpF-dCas9, pgRNA-pSC101bb), CAR025-CrtZ-*CK*(CAR025 co-transformed with plasmids pSC101-CrtZ,pGlpF-dCas9, pgRNA-N20).

a

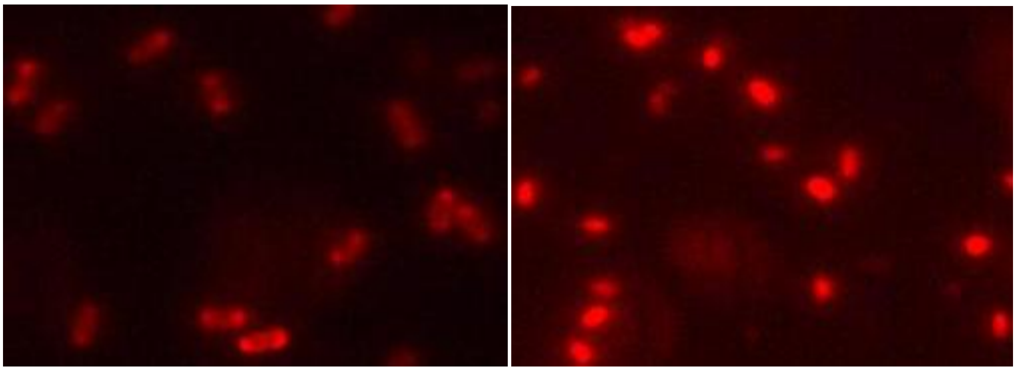

b

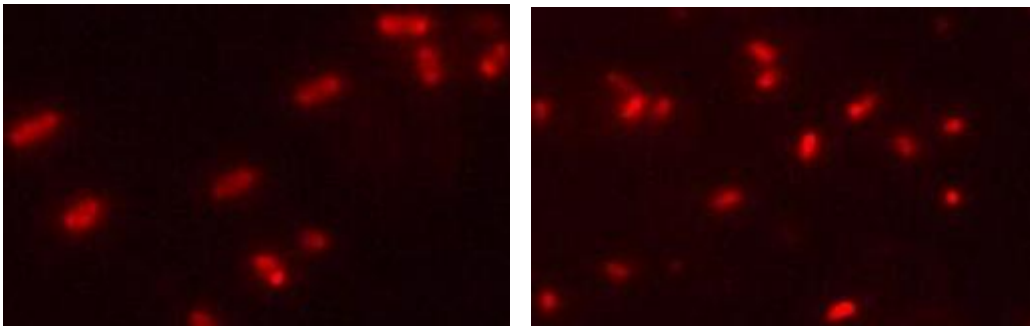

Figure S3 The fluorescence microscopy of cells with Cas9-Lag targeted plasmids expressing RFP fusion. a) The fluorescence microscopy images of CAR025-CrtZ-RFP and it's control strain CAR025-CrtZ-RFP-*CK*; b) The fluorescence microscopy images of CAR025-CrtW-RFP and it's contol strain CAR025-CrtW-RFP-*CK*.

pTrc-GlpF-GGGS-dCas9

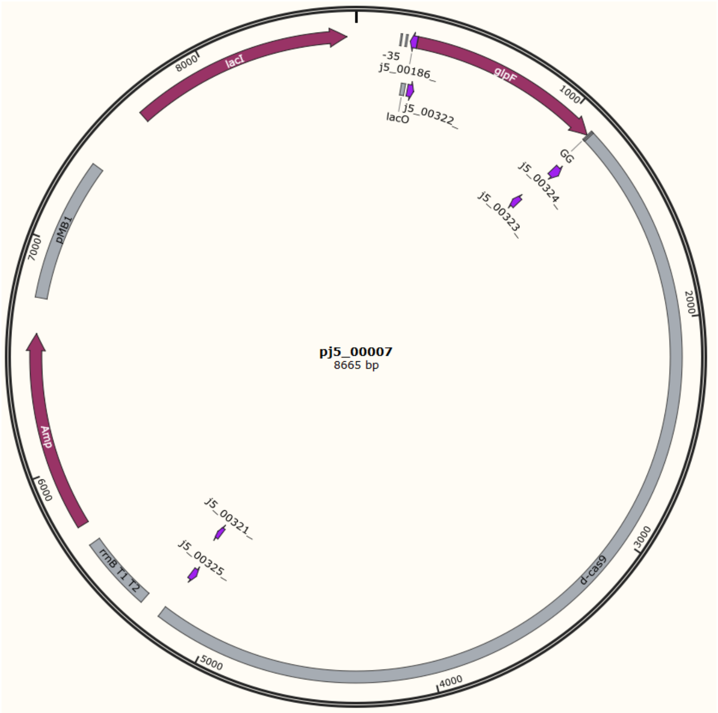

>*GlpF* sequence

ATGAGTCAAACATCAACCTTGAAAGGCCAGTGCATTGCTGAATTCCTCGGT
ACCGGGTTGTTGATTTTCTTCGGTGTGGGTTGCGTTGCAGCACTAAAAGTC
GCTGGTGCGTCTTTTGGTCAGTGGGAAATCAGTGTCATTTGGGGACTGGGG
GTGGCAATGGCCATCTACCTGACCGCAGGGGTTTCCGGCGCGCATCTTAAT
CCCGCTGTTACCATTGCATTGTGGCTGTTTGCCTGTTTCGACAAGCGCAAA
GTTATTCCTTTTATCGTTTCACAAGTTGCCGGCGCTTTCTGTGCTGCGGCTTT
AGTTTACGGGCTTTACTACAATTTATTTTTCGACTTCGAGCAGACTCATCAC
ATTGTTCGCGGCAGCGTTGAAAGTGTTGATCTGGCTGGCACTTTCTCTACTT
ACCCTAATCCTCATATCAATTTTGTGCAGGCTTTCGCAGTTGAGATGGTGAT
TACCGCTATTCTGATGGGGCTGATCCTGGCGTTAACGGACGATGGCAACGG
TGTACCACGCGGCCCTTTGGCTCCCTTGCTGATTGGTCTACTGATTGCGGTC
ATTGGCGCATCTATGGGCCCATTGACAGGTTTTGCCATGAACCCAGCGCGT
GACTTCGGTCCGAAAGTCTTTGCCTGGCTGGCGGGCTGGGGCAATGTCGC
CTTTACCGGCGGCAGAGACATTCCTTACTTCCTGGTGCCGCTTTTCGGCCCT
ATCGTTGGCGCGATTGTAGGTGCATTTGCCTACCGCAAACTGATTGGTCGCC
ATTTGCCTTGCGATATCTGTGTTGTGGAAGAAAAGGAAACCACAACTCCTT
CAGAACAAAAAGCTTCGCTGTAA

pgRNA plasmid

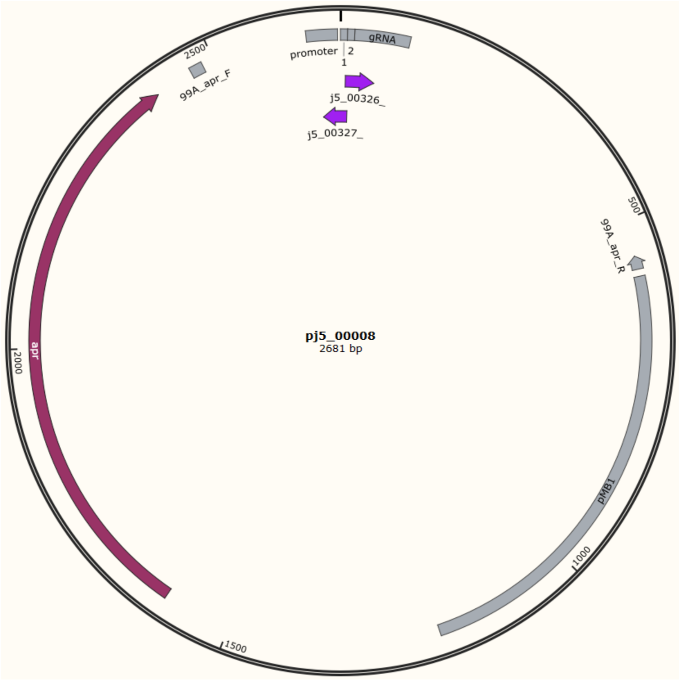

pBAD-Rfp

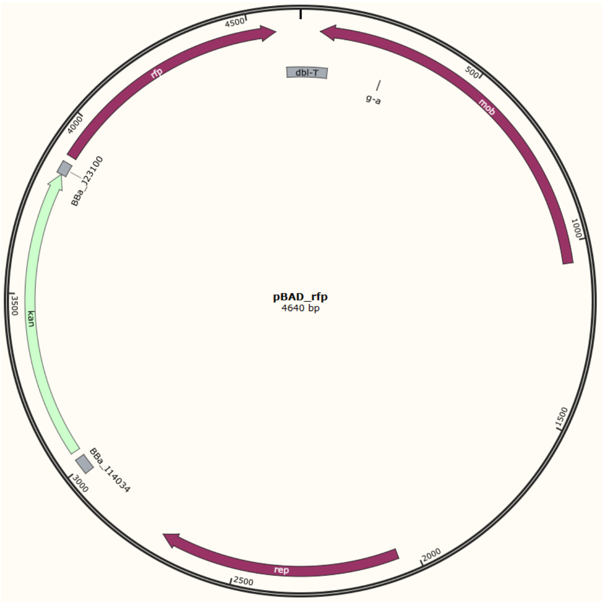

>*rfp* sequence

ATGGCGAGTAGCGAAGACGTTATCAAAGAGTTCATGCGTTTCAAAGTTCGT
ATGGAAGGTTCCGTTAACGGTCACGAGTTCGAAATCGAAGGTGAAGGTGA
AGGTCGTCCGTACGAAGGTACCCAGACCGCTAAACTGAAAGTTACCAAAG
GTGGTCCGCTGCCGTTCGCTTGGGACATCCTGTCCCCGCAGTTCCAGTACG
GTTCCAAAGCTTACGTTAAACACCCGGCTGACATCCCGGACTACCTGAAAC
TGTCCTTCCCGGAAGGTTTCAAATGGGAACGTGTTATGAACTTCGAAGACG
GTGGTGTTGTTACCGTTACCCAGGACTCCTCCCTGCAAGACGGTGAGTTCA
TCTACAAAGTTAAACTGCGTGGTACCAACTTCCCGTCCGACGGTCCGGTTA

TGCAGAAAAAAACCATGGGTTGGGAAGCTTCCACCGAACGTATGTACCCG
GAAGACGGTGCTCTGAAAGGTGAAATCAAAATGCGTCTGAAACTGAAAGA
CGGTGGTCACTACGACGCTGAAGTTAAAACCACCTACATGGCTAAAAAAC
CGGTTCAGCTGCCGGGTGCTTACAAAACCGACATCAAACTGGACATCACCT
CCCACAACGAAGACTACACCATCGTTGAACAGTACGAACGTGCTGAAGGT
CGTCACTCCACCGGTGCTTAA

PYL501 plasmid

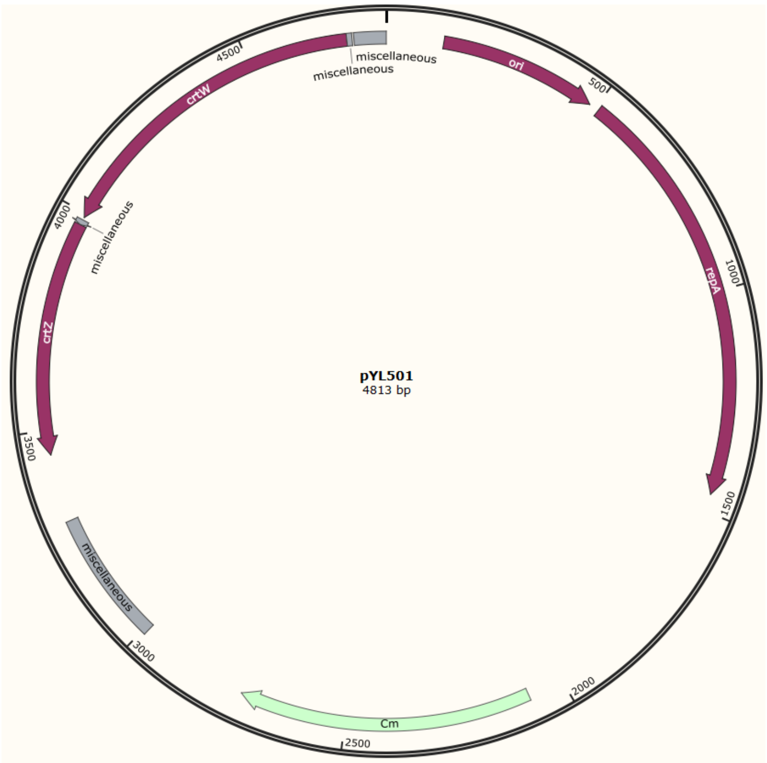

>*CrtW* sequence
ATGACCGCCGCAGTCGCAGAACCGCGTATTGTCCCGCGTCAAACCTGGAT
TGGCCTGACCCTGGCTGGTATGATTGTTGCTGGCTGGGGCTCCCTGCATG
TTTATGGTGTCTACTTTCATCGTTGGGGCACCAGCTCTCTGGTGATTGTT
CCGGCAATCGTCGCTGTGCAGACGTGGCTGTCAGTGGGTCTGTTTATTGT
TGCGCATGATGCCATGCACGGTTCGCTGGCGCCGGGTCGTCCGCGTCTGA
ACGCGGCCGTGGGCCGCCTGACCCTGGGCCTGTATGCCGGTTTTCGTTTC
GATCGCCTGAAAACCGCACATCACGCTCATCACGCAGCTCCGGGTACGGC
AGATGACCCGGACTTTTATGCACCGGCTCCGCGTGCTTTTCTGCCGTGGT
TCCTGAACTTTTTCCGTACCTACTTCGGCTGGCGCGAAATGGCAGTCCTG
ACGGCTCTGGTTCTGATCGCACTGTTTGGTCTGGGTGCACGTCCGGCAAA
TCTGCTGACCTTCTGGGCAGCACCGGCACTGCTGAGCGCACTGCAGATGT
TTACCTTCGGCACGTGGCTGCCGCATCGTCACACCGATCAACCGTTTGCA
GACGCACATCACGCACGTAGTTCCGGTTACGGTCCGGTTCTGTCTCTGCT
GACGTGCTTCCATCTGGGTCGTCACCATGAACATCATCTGACCCCGTGGC
GTCCGTGGTGGCGTCTGTGGCGTGGTGAATCGTAA

>*CrtZ* sequence

ATGTTGTGGATTTGGAATGCCCTGATTGTTCTGGTCACTGTTATCGGAATGG
AGATAACGGCTGCACTGGCGCACAGATACATTATGCATGGCTGGGGTTGGG
GCTGGCATCTGTCACATCATGAACCGCATAAAGGCTGGTTTGAGGTTAATG
ACCTCTATGCCGTAGTGTTCGCCGCTCTGTCGATTTTGCTCATTTATCTGGGC
AGCACGGGTGTCTGGCCCTTGCAGTGGATCGGCGCGGGTATGACGCTCTAC
GGCCTGCTCTATTTTATTGTGCATGATGGCCTGGTACATCAGCGCTGGCCAT
TCCGCTATGTTCCGCGCCGGGGTTATTTACGCAGGCTTTACATGGCGCACCG
CATGCATCATGCGGTACGAGGCAAAGAGGGCTGTGTCTCGTTTGGCTTTCT
TTACGCGCCGCCGCTGTCAAAACTGCAGGCGACGCTGCGAGAACGTCATG
GCGTTAAACGGGGCGCTGCCAGAGATCAGCTGAGCGTGGAGCGTGACGCG
CCACCCGGGAAGTAA
